# Magnetic resonance elastography in evaluation of liver fibrosis in children with chronic liver disease

**DOI:** 10.1186/s13244-023-01390-0

**Published:** 2023-02-28

**Authors:** Duygu Demirtaş, Emre Ünal, İlkay S. İdilman, Zuhal Akçören, Mehmet Akif Göktaş, Meryem Seda Boyraz, Sevilay Karahan, Diclehan Orhan, Mithat Haliloğlu, Muşturay Karçaaltıncaba, Hasan Özen

**Affiliations:** 1 Division of Pediatric Gastroenterology, Hepatology and Nutrition, Faculty of Medicine, Hacettepe University, Ankara, Turkey; 2 Department of Radiology, Faculty of Medicine, Hacettepe University, Ankara, Turkey; 3 Department of Pediatric Pathology, Faculty of Medicine, Hacettepe University, Ankara, Turkey; 4 Department of Biostatistics, Faculty of Medicine, Hacettepe University, Ankara, Turkey

**Keywords:** Ishak scoring system, Liver stiffness, Magnetic resonance elastography, Spleen stiffness

## Abstract

**Background:**

Magnetic resonance elastography (MRE) has been used to stage liver fibrosis in adults. We aimed to assess the agreement between the Ishak scoring system and magnetic resonance elastography-measured liver stiffness (MRE-LS) in children. This study included all the children who underwent abdominal MRE and liver biopsies between February 2018 and January 2021. The correlation between MRE-LS and Ishak fibrosis stage, MRE parameters, and clinical and biochemical markers affecting this relationship was investigated.

**Results:**

A total of 52 patients (31 male; a median age of 11.8 years) were included in the study. The MRE-LS values were significantly different between Ishak fibrosis stages (*p* = 0.036). With a cut-off value of 2.97 kilopascals, MRE-LS had sensitivity, specificity, PPV, NPV and accuracy values of 90.9%, 82.9%, 58.8%, 97.1%, and 84.6%, respectively, for differentiating mild/moderate fibrosis (F0, 1, 2, 3) from severe fibrosis (F ≥ 4). Although MRE-LS was moderately correlated with Ishak fibrosis score and histological activity index and weakly correlated with aspartate aminotransferase, hepatic steatosis, and R2*, only Ishak fibrosis score was a significant predictor of MRE-LS. MRE-measured spleen stiffness was weakly correlated with the Ishak fibrosis score.

**Conclusions:**

MRE has high sensitivity and specificity for evaluating liver fibrosis in children. MRE may be used to evaluate liver fibrosis in pediatric patients.

**Supplementary Information:**

The online version contains supplementary material available at 10.1186/s13244-023-01390-0.

## Key points

1. MRE has high sensitivity and specificity in evaluating liver fibrosis in children.
2. MRE-LS is significantly correlated with histological Ishak fibrosis stage.
3. Comparing to other parameters, only MRE-LS is a predictor of fibrosis stage.
4. In the future, MRE may replace biopsy to evaluate liver fibrosis.

**Supplementary Information:**

The online version contains supplementary material available at 10.1186/s13244-023-01390-0.

## Background

Liver biopsy is considered the gold standard to assess the presence and severity of fibrosis and may provide useful information about the underlying etiology of chronic liver disease (CLD). However, complications, such as bleeding and organ damage, are possible. Moreover, only a small portion of the liver can be sampled with a liver biopsy. As a result, the sample size and distribution of the disease throughout the liver parenchyma can affect diagnostic accuracy. Interobserver variability is another limitation of histological assessment [1]. For these reasons, non-invasive methods have been researched and used with increasing frequency to assess the degree of liver fibrosis [2]. Ultrasound-based elastography and magnetic resonance elastography (MRE) represent larger liver samples and may provide a more accurate assessment of liver fibrosis. Ultrasound-based elastography relies more on operator skill and can be unreliable in obese patients or patients with ascites [3, 4]. MRE is more accurate than ultrasound-based elastography in identifying liver fibrosis in adults [5–8]. Although ultrasound-based elastography has been used as a non-invasive tool for staging liver fibrosis in children with CLD, only a few studies have compared MRE with liver biopsy [4, 9, 10].

There are several histopathological liver fibrosis staging systems, including the Metavir, Batts-Ludwig, International Association for the Study of the Liver, Knodell, and Ishak (modified Knodell) fibrosis scores [11]. Except for the Ishak scoring system, the others evaluate the severity of fibrosis in five stages. Because it includes more stages (0–6) and can clearly distinguish incomplete cirrhosis from established cirrhosis, the Ishak fibrosis score has a higher sensitivity for fibrosis assessment [11, 12].

Although studies have investigated the compatibility of ultrasound-based elastography with the Ishak fibrosis scoring system, no study has been performed with MRE. The primary aim of this study was to evaluate the agreement between the Ishak scoring system and MRE-measured liver stiffness (MRE-LS) for assessing the degree of liver fibrosis. The secondary aims were to evaluate the correlation between the Ishak scoring system and MRE-measured spleen stiffness (MRE-SS) and the factors that may affect liver stiffness measurement with MRE.

## Methods

All children younger than 18 years who both underwent abdominal MRE and liver biopsy between February 2018 and January 2021 at Hacettepe University Children’s Hospital, Department of Pediatric Gastroenterology, were included in the study. Ishak fibrosis score was accepted as the gold standard for liver fibrosis assessment, and the concordance of MRE-LS with Ishak fibrosis score was evaluated retrospectively. Age, sex, weight, height, body mass index (BMI), BMI Z-scores, alanine aminotransferase (ALT) and aspartate aminotransferase (AST) levels were recorded. The radiologist and pathologist were blinded to the pathological and imaging data, respectively. The histological fibrosis stage and activity index (HAI) were determined for each biopsy specimen according to the Ishak scoring system: 0, no fibrosis, 5 indicating incomplete cirrhosis, and 6, cirrhosis. The HAI was graded from 0 to 18, with 0 indicating no necroinflammation and 18 indicating the highest possible score for necroinflammation (minimal for 1–3, mild for 4–8, moderate for 9–12, and severe for 13–18 points) [11]. Alanine aminotransferase and AST levels > 40 IU/L were considered as high.

Magnetic resonance imaging was performed using a 1.5-T MR system (Magnetom Aera^®^, Siemens Healthcare, Erlangen, Germany) and a 30-channel phased-array body coil. The parameters of the MRE were as follows: TR/TE, 50 ms/21.41 ms, flip angle 25°, section thickness 50 mm, and field of view 350 × 350 mm^2^ with a passive driver frequency of 60 Hz. T1, T2, and T2* mapping were also performed. Corrected T1 (cT1) relaxation values were calculated using the formula “T1 − 420 + 20 × T2*” over T2* values (13).

Regions of interests (ROIs) were drawn as geographic areas guided by the magnitude image to include the liver parenchyma while excluding major vessels using a workstation (Syngo. via VB10; Siemens Medical Solutions). ROIs were copied to the stiffness maps and corrected according to confidence map images. MRE-LS and MRE-SS were calculated by averaging the stiffness obtained from each slice of the MRE sequence. T1 relaxation, T2 relaxation, and T2* values were obtained from the corresponding map images with a large freehand ROI, excluding lesions, large vessels, liver margins, and artefacts from the right lobe of the liver. Hepatic steatosis was also measured and defined as 5% or more fat of hepatocytes: < 5% no steatosis, 5–33% score 1, 34–66% score 2, and > 66% score 3 (7).

The Institutional Ethics Committee approved the study.

### Statistical analysis

An experienced faculty biostatistician performed the statistical analyses using SPSS for Windows Version 22.0 statistical package (Chicago, IL). Continuous variables were summarized as mean ± standard deviation when normally distributed or median (25–75th percentiles; interquartile range-IQR) when they did not follow a normal distribution. Categorical variables are presented as frequencies and percentages.

Mann–Whitney U test and Kruskal–Wallis test were used to compare differences between two and more than two independent groups, respectively. The patients were divided into two groups according to the Ishak fibrosis score: Group 1, no fibrosis or moderate fibrosis (F0, 1, 2, 3) and Group 2, severe fibrosis or cirrhosis (F4, 5, 6).

The correlation between continuous and ordinal variables was determined using the Spearman correlation coefficient (95% confidence interval [CI]). The chi-square test or Fisher’s exact test was used to compare frequencies or proportions between groups.

Correlations between MRE-LS and MRE-SS, T1, T2, T2*, cT1, R2*, HAI, liver fat content, AST value, BMI Z-scores, and Ishak fibrosis stages were calculated. Correlation strength of 0–0.20 was considered very weak; 0.21–0.40 weak; 0.41–0.60 moderate; 0.61–0.80 strong, and 0.8−  < 1.00 very strong. The effect of the variables correlated with MRE-LS and MRE-SS on the relationship between Ishak stages and MRE-LS, or MRE-SS, was tested using partial correlation. Additionally, the significance of the variables correlated with MRE-LS or MRE-SS for liver or spleen stiffness was tested using multiple linear regression analysis.

Receiver operating characteristic (ROC) curve analyses were performed to detect the optimal cut-off values and area under the ROC curve (AUROC) for liver stiffness and spleen stiffness. Cut-off values were determined using the highest Youden index. Sensitivity, specificity, positive predictive value (PPV), negative predictive value (NPV), and total accuracy were calculated at these cut-off points. The significance value was set at a two-tailed *p*-value of < 0.05.

## Results

All continuous variables, except the BMI Z-scores, followed an abnormal distribution. A total of 52 patients, 31 male (59.6%) were included in the study. The majority of the biopsies were done for diagnosis of liver disease etiology. The median age was 11.8 years (IQR 8.0–15.6). The median time between MRE and liver biopsy was one day (IQR 0.0–2.0). Table 1 shows the patients’ demographic and clinical characteristics.

**Table 1:** Patient demographics and characteristics

Sex	
Male	31 (59.6%)
Female	21 (40.4%)
Age (y)	
Median (25–75%)	11.8 (8.0–15.6)
Time interval between	
MRI and liver biopsy, day, median (25–75%)	1 (0–2)
BMI *Z*-score, mean ± SD (range)	0.1921 ± 1.4983 (− 3.29 − + 3.90)
> + 2 n (%)	6 (11.5%)
< − 2 n (%)	2 (3.8%)
Etiology of the liver disease—no. (%)	
Chronic hepatitis of uncertain etiology	14 (26.9%)
Autoimmune hepatitis	9 (17.3%)
Chronic viral hepatitis	7 (13.5%)
Genetic/metabolic disorders	7 (13.5%)
Cirrhosis of different etiology	5 (9.6%)
Others	10 (19.2%)
Ishak fibrosis stages, median (25–75%)	2.0 (1.0–3.0)
Stage	
0	3 (5.8%)
1	15 (28.8%)
2	10 (19.2%)
3	13 (25.0%)
4	6 (11.5%)
5	1 (1.9%)
6	4 (7.7%)
Histologic activity index, median (25–75%)	3.0 (2.0–5.0)
No	1 (1.9%)
Minimal (1–3 points)	28 (53.8%)
Mild (4–8 points)	19 (36.5%)
Moderate (9–12 points)	4 (7.7%)
Severe (13–18 points)	0 (0%)
Fat/steatosis (n = 51), median (25–75%)	2.9 (2.2–10.9%)
0 (< 5%)	35 (67.3%)
1 (5–33%)	15 (28.8%)
2 (34–66%)	1 (1.9%)
3 (> 66%)	0 (0.0%)
ALT IU/L, median (25–75%)	69.5 (31.0–176.3)
> 40 IU/L (%)	36 (69.2%)
AST IU/L, median (25–75%)	79.0 (32.5–183.75)
> 40 IU/L (%)	35 (67.3%)

MRE-SS could not be obtained from three patients as insufficient spleen parenchyma was included in the MR elastography images. Liver fat content and R2* could not be obtained from one patient as a result of obvious motion artifacts. MRE-LS values (kilopascal-kPa) were significantly different between Ishak fibrosis stages (*p* = 0.036), while MRE-SS values did not differ between fibrosis stages (*p* = 0.712). Liver stiffness on MRE was significantly higher in Group 2 than in Group 1 (3.99 ± 1.05 kPa vs. 2.50 ± 0.64, *p* < 0.001). Although statistically insignificant, spleen stiffness was also higher in Group 2 (6.26 ± 4.25 kPa vs. 3.70 ± 2.01 kPa, *p* = 0.065).

MRE-LS and spleen stiffness were weakly correlated (r_s_ = 0.297, *p* = 0.038). MRE-LS was moderately correlated with the Ishak fibrosis score (r = 0.553, *p* < 0.001) and HAI (r = 0.406, *p* = 0.003), and weakly correlated with AST (r = 0.364, *p* = 0.008), steatosis (r = − 0.330, *p* = 0.018), and R2* (r = − 0.326, *p* = 0.02). The abnormal AST and higher HAI grade proportions were significantly higher in patients with higher Ishak fibrosis scores (*p* = 0.004 and *p* = 0.01, respectively). Only Ishak fibrosis score was a significant predictor of MRE-LS values in the multiple linear regression analysis. MRE-SS values were weakly correlated with Ishak fibrosis scores (r = 0.357, *p* = 0.012) (Additional file 1). After controlling for the effects of HAI, hepatic steatosis, AST level, and R2*, the strength and direction of the relationship between MRE-LS and Ishak fibrosis stage did not change (r = 0.496, *p* < 0.001). In contrast, the correlation strength between MRE-SS and Ishak fibrosis stages increased (r = 0.452, *p* = 0.002).

**Fig. 1:**
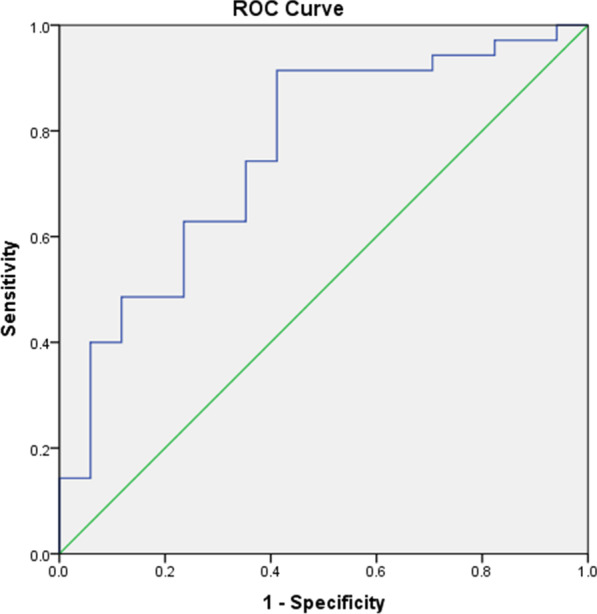
Receiver operating characteristic curve for differentiation of Ishak stage 0–1 from stage 2 or higher. *Area Under Curve 0.760 (95% CI 0.618–0.901) (*p* = 0.003)

The diagnostic cut-off values of liver stiffness and spleen stiffness on MRE to differentiate each liver fibrosis stage (F0 vs. F1–6, F0–1 vs. F2–6, F0–3 vs. F4–6, F0–4 vs. F5–6, and F0–5 vs. F6) are given in Table 2. With a cut-off value of 2.65 kPa, the AUC was 0.633 (*p* = 0.302), and its sensitivity, specificity, PPV, NPV, and accuracy for differentiating no fibrosis from any fibrosis were 40.8%, 100%, 100%, 9.4%, and 44.2%, respectively. The same parameters for spleen stiffness were 0.638 (*p* = 0.526), 100%, 33.3%, 95.8%, 100%, and 95.9%, respectively. When no fibrosis-minimal fibrosis (F0–1) was compared with a higher degree of fibrosis (F ≥ 2), with a cut-off value of 2.14 kPa, the AUC was 0.760 (*p* = 0.003), and its sensitivity, specificity, PPV, NPV, and accuracy were 91.4%, 58.8%, 82.1%, 76.9%, and 80.8%, respectively (Fig. 1. Receiver operating characteristic curve for differentiation of Ishak stage 0–1 from stage 2 or higher). The same parameters for spleen stiffness were 64.2% (*p* = 0.112), 73.5%, 53.3%, 78.1%, 47.1%, and 67.3%, respectively.

**Table 2:** Diagnostic performance of liver stiffness and spleen stiffness to detect hepatic fibrosis

	Fibrosis stage	n	Cut-off (kPa)	AUC (95% CI)	p	Sensitivity (95% CI)	Specificity (95% CI)	PPV (95% CI)	NPV (95% CI)	Accuracy
Liver stiffness (*n* = 52)	> F0	49	2.65	0.63 (0.49–0.85)	0.302	40.8 (27–55.8)	100 (29.2–100)	100 (83.2–100)	9.4 (2–25)	44.2
	> F1	35	2.14	0.76 (0.62–0.87)	< 0.001	91.4 (76.9–98.2)	58.8 (32.9–81.6)	82.1 (66.5–92.5)	76.9 (46.2–95)	80.8
	> F2	24	2.8	0.74 (0.56–0.85)	< 0.001	58.3 (36.6–77.9)	85.7 (67.3–96)	77.8 (52.4–93.6)	70.6 (52.5–84.9)	73.1
	> F3	11	2.97	0.90 (0.79–0.97)	< 0.001	90.9 (58.7–99.8)	82.9 (67.9–92.8)	58.8 (32.9–81.6)	97.1 (85.1–99.9)	84.6
	> F4	5	3.14	0.89 (0.78–0.96)	< 0.001	100 (47.8–100)	80.9 (66.7–90.9)	35.7 (12.8–64.9)	100 (90.7–100)	82.7
	> F5	4	3.14	0.86 (0.74–0.94)	< 0.001	100 (39.8–100)	79.2 (65–89.5)	28.6 (8.4–58.1)	100 (90.7–100)	80.8
Spleen stiffness (*n* = 49)	> F0	46	0.5	0.64 (0.49–0.77)	0.526	100 (92.3–100)	33.3 (0.8–90.6)	95.8 (85.7–99.5)	100 (2.5–100)	95.9
	> F1	34	2.87	0.64 (0.49–0.77)	0.112	73.5 (55.6–87.1)	53.3 (26.6–78.7)	78.1 (60–90.7)	47.1 (23–72.2)	67.3
	> F2	23	3.38	0.69 (0.55–0.82)	0.010	73.9 (51.6–89.8)	57.7 (36.9–76.6)	60.7 (40.6–78.5)	71.4 (47.8–88.7)	65.3
	> F3	11	6.26	0.68 (0.54–0.81)	0.083	45.5 (16.7–76.6)	94.7 (82.3–99.4)	71.4 (29–96.3)	85.7 (71.5–94.6)	83.7
	> F4	5	8.66	0.82 (0.69–0.92)	0.002	60 (14.7–94.7)	97.7 (88–99.9)	75 (19.4–99.4)	95.6 (84.9–99.5)	93.9
	> F5	4	3.42	0.78 (0.64–0.89)	0.030	100 (39.8–100)	48.9 (33.7–64.2)	14.8 (4.2–33.7)	100 (84.6–100)	53.1

**Fig. 2:**
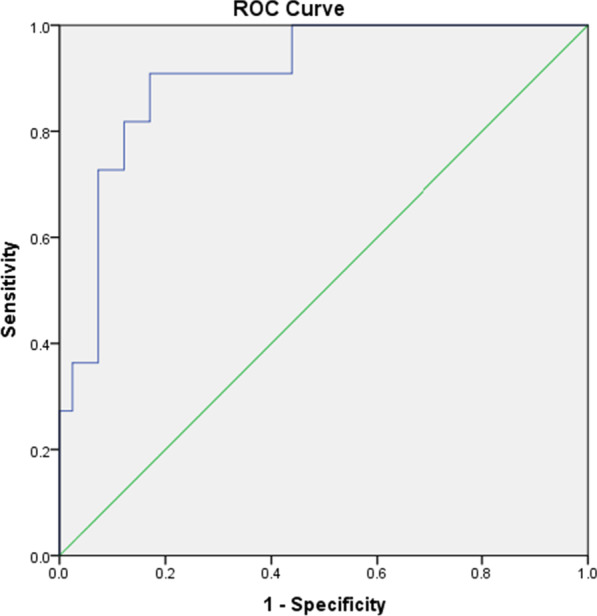
Receiver operating characteristic curve for differentiation of Group 1 (Ishak stage 0–1-2–3) from Group 2 (Ishak stage 4–5–6). *Area Under Curve 0.905 (95% CI 0.814–0.996) (*p* < 0.001)

When comparing Groups 1 and 2, with a cut-off value of 2.97 kPa for the liver, the AUC was 0.905 (*p* < 0.001), and the sensitivity, specificity, PPV, NPV, and accuracy were 90.9%, 82.9%, 58.8%, 97.1%, and 84.6%, respectively [Fig. 2. Receiver operating characteristic curve for differentiation of Group 1 (Ishak stage 0–1–2–3) from Group 2 (Ishak stage 4–5–6)]. With a cut-off value of 6.26 kPa for MRE-SS, the AUC, sensitivity, specificity, PPV, NPV, and accuracy for differentiating the same groups were 0.684 (*p* = 0.083), 45.5%, 94.7%, 71.4%, 85.7%, and 83.7%, respectively. The AUC values for T1, T2, T2*, and cT1 were not statistically significant.

When patients were divided into two groups with fibrosis stages 0–1–2–3–4 and 5–6 (cirrhosis) according to the Ishak scoring system, with a cut-off value of 3.15 kPa for the liver, the AUC was 0.898 (*p* < 0.001) and its sensitivity, specificity, PPV, NPV, and accuracy were 100.0%, 80.9%, 35.7%, 100.0%, and 82.7%, respectively. With a cut-off value of 8.26 kPa for spleen, AUC was 0.825 (*p* = 0.002), and its sensitivity, specificity, PPV, NPV, and accuracy were 60.0%, 97.7%, 75.0%, 95.6%, and 93.9%, respectively. The AUC values for T1, T2, T2*, and cT1 were not statistically significant. However, there were only five patients in the second group.

Only six patients had a BMI Z-score of >  + 2. Those with a BMI Z-score >  + 2 had a higher liver fat ratio of ≥ 5% (hepatic steatosis) than those with a BMI Z-score ≤ 2 (*p* = 0.047), and the median liver fat ratio (2.7% vs. 17.6%, *p* = 0.012) and median R2* value (25.3 vs 30.6 kPa, *p* = 0.009) were higher in obese patients. The other variables, including MRE-LS and MRE-SS, were not significantly different between the two groups. Nevertheless, there were no obese patients with Ishak fibrosis stage ≥ 4.

## Discussion

MRE has a very high NPV for distinguishing between mild/moderate fibrosis (F0, 1, 2, 3) and severe fibrosis (F ≥ 4). It can be concluded that children without fibrosis in MRE are less likely to have significant liver fibrosis, and in these children, liver biopsy may not be performed solely to evaluate liver fibrosis. In addition, MRE had 100% sensitivity and 80.8% specificity with a liver stiffness cut-off of 3.15 kPa for detecting Ishak stages 5–6 (incomplete and probable or definite cirrhosis) fibrosis (Table 2).

Only the Ishak fibrosis score was a significant predictor of MRE-LS when all clinical and laboratory parameters were evaluated. These results agree with previous studies reporting that both liver and spleen stiffness correlate with the degree of liver fibrosis [13].

Although we showed that MRE-SS increased as the stage of Ishak fibrosis increased, the findings were not as significant as those in liver measurements. Using a spleen stiffness cut-off of 6.26 kPa, the AUC for detecting Ishak stages 4–5–6 fibrosis was 0.684 (Table 2).

The value of normal MRE-LS in children is controversial. While normal liver stiffness values were shown to be lower [14] or similar [15] in children compared to adults, a recent study with healthy children aged between 8 and 17 years showed that mean liver stiffness was significantly higher than reported values for healthy adults (*p* < 0.001) [16]. In our study, the number of patients with no fibrosis is only three. When the patients with no fibrosis-minimal fibrosis (F0–1) were compared with those with F ≥ 2, with a cut-off value of 2.14 kPa, the AUC was 76% (*p* < 0.001), and its sensitivity and specificity were 91.4% and 58.8%, respectively. More studies are needed to clarify normal liver stiffness values in healthy children.

In a study involving 68 children aged 2–12 years with various CLD [17], it was found that shear wave speed (SWS) was significantly higher in patients with F4-6 fibrosis than in those with F0–3 fibrosis (*p* = 0.02). Similar to our study, there was no correlation between BMI and liver stiffness (*p* = 0.849), and the fibrosis stage was the only significant predictor of SWS. In 2020, Dardanelli et al. [18] showed a good correlation between two-dimensional (2D) shear wave elastography (SWE) measurement and the Ishak fibrosis stage in 213 children with CLD. They also showed that 2D SWE could differentiate mild (F1–2) vs. moderate (F3–4) and severe (F5–6) vs. milder fibrosis, with a sensitivity (and specificity) of 92% (86%) and 94% (90%), respectively. Similar to these studies, MRE-LS and MRE-SS correlated with the liver fibrosis stage, and the liver fibrosis stage was the only predictor of MRE-LS in our study.

MRE is capable to evaluate the whole liver in a short acquisition time in comparison with US elastography. Moreover, MRE is less operator dependent than US elastography and repeatable for the diagnosis and quantitative staging of liver fibrosis [19]. It is also shown that MRE has the highest accuracy for stage 4 fibrosis (cirrhosis, Brunt grading system for liver fibrosis) detection and intra-inter observer reproducibility [20].

Studies evaluating the agreement between histological fibrosis stages, especially with the Ishak scoring system and MRE-LS in children, are still scarce. The first study evaluating liver fibrosis with MRE was published by Binkovitz et al. in 2012 in seven children with various CLD [21]. They reported that the presence or absence of liver fibrosis was demonstrated with MRE with a sensitivity of 98% and specificity of 99%, with a normal cut-off value of < 2.93 kPa.

In a study comparing six cystic fibrosis patients with cirrhosis (age range 7–44 years) and four healthy individuals, a cut-off value of > 3.38 kPa was both 100% sensitive and specific for distinguishing cirrhosis (32). The correlation between fibrosis stage and MRE measurements has not been reported in these studies.

In previous studies comparing MRE and liver biopsy in children [4, 9, 10], fibrosis was staged as 0–4, with 4 indicating cirrhosis. To the best of our knowledge, our study is the first to compare MRE-LS with Ishak score to evaluate liver fibrosis in pediatric patients. In addition, the time between MRE and liver biopsy was shorter than that reported in previous studies.

Similar to our study, in their prospective, exploratory study, Gharib et al. [22] showed a significant correlation between MRE-LS values and the Ishak fibrosis stage in 23 adult patients with HCV infection (r = 0.71, *p* = 0.004). In a pilot study by Xanthakos et al. [10], liver fibrosis was evaluated using MRE in 35 children (median age 13 years) with CLD. Twenty-seven of them had nonalcoholic fatty liver disease (NAFLD) (22 with nonalcoholic steatohepatitis [NASH]), and the remaining patients had different CLD. The median time interval between scan and biopsy was 1.5 months (IQR “− 0.5” and 4 months). Fibrosis was staged from 0 to 4. Although they did not report the BMI Z-scores of the patients, the median BMI percentile of the patients was 99.2, indicating severe obesity. Similar to our study, they showed that liver stiffness increased with advancing fibrosis stages. MRE had 88% sensitivity and 85% specificity at a cut-off of 2.71 kPa for detecting significant fibrosis (stages ≥ 2) with an AUROC of 0.92 (95% CI 0.79–1.00;  = 0.02). The markedly high BMI range and small sample size may be limitations of that study.

In a two-center study by Schwimmer et al. (6), liver fibrosis was evaluated using MRE in 87 children with NAFLD (49 also had NASH) and 3 without NAFLD. The mean age was 13.1 ± 2.1 years, and 89 of them had a BMI Z-score > 2. The severity of liver fibrosis was graded from 0 to 4 as follows: no fibrosis (54 patients), stage 1 (27 patients), stage 2 (6 patients), stage 3 (5 patients), and stage 4 (1 patient). Advanced fibrosis was defined as a stage ≥ 3. The median liver stiffness was 2.35 kPa, and measurements similar to those in our study were significantly correlated with the fibrosis stage (*p* < 0.001). In an automated analysis, MRE had 33.3% sensitivity, 90.5% specificity, PPV 20.0%, NPV 95.0%, and total accuracy of 86.7% with a liver stiffness cut-off of 3.33 kPa for differentiating advanced fibrosis [4].

Iron overload in the liver tissue is the most common cause of technical failure in MRE. However, steatohepatitis, hepatic vascular congestion, cholestasis, amyloidosis, and fasting can also affect liver stiffness [6, 23, 24]. In their study involving 81 children (6–18 years) with AIH, Janowski et al. [25] showed that the degree of inflammation could affect MRE measurement, and iron-cT1, an objective composite biomarker of fibro-inflammation, may help counteract inflammation [25]. Baseline cT1 correlates with histological inflammation and fibrosis in patients with AIH. In our study, neither T1 nor cT1 correlated with histological activity. Nevertheless, there were only seven patients with AIH in our study, and the HAI was quite low (median 3.0).

However, the influence of liver steatosis on liver stiffness measurements is contradictory. There seems that fat confounds MRE-LS values, particularly at the early stages of fibrosis [6, 8, 9]. Trout et al. [9] evaluated liver stiffness using MRE in 86 children (median age 14.2 years; range, 0.3–20.6 years) with a spectrum of liver diseases. They evaluated liver fibrosis according to Ludwig stages (stage 0–4) and defined steatosis as the presence of lipid in ≥ 5% of hepatocytes. Fifty-one patients (59.3%) had Ludwig stage ≥ 2 fibrosis, and 44 patients (51.2%) had liver steatosis. They found that steatosis significantly decreased the ROC curve for Ludwig stage 0–1 vs. ≥ 2 (0.53 vs. 0.82, *p* = 0.014). They concluded that MRE performs significantly better for distinguishing the fibrosis stage in patients without steatosis than in those with steatosis. In our study, 67.3% of the patients had no steatosis, and there was only one patient with grade 2 steatosis. Steatosis was weakly negatively correlated with MRE measurement, and its effect disappeared when evaluating with the other confounding variables. Therefore, we could not compare our results with those reported by Trout et al. [9].

Our study is not without limitations. The most important limitations of our study are the variety of liver diseases and the small number of patients without fibrosis (F0) and with severe (F4–6) fibrosis. The degree, pattern, distribution, and extent of fibrosis can vary depending on the etiology of CLD, and thresholds used to determine the degree of fibrosis may also vary depending on the etiology [26, 27]. Therefore, studies with more patients should evaluate the agreement of MRE and the Ishak scoring system in different etiologies.

## Conclusion

MRE has high sensitivity and specificity for evaluating liver fibrosis in children. MRE may be used to evaluate liver fibrosis in children instead of liver biopsy. To the best of our knowledge, our study is the first to compare MRE-LS with Ishak score to evaluate liver fibrosis in pediatric patients.

## Supplementary Information

**Additional file 1**. Correlations between MRE-measured liver and spleen stiffness and clinical, laboratory and other MRE parameters

## Data Availability

The datasets used and/or analysed during the current study are available from the corresponding author on reasonable request.

## Abbreviations

2D: Two-dimensional
ALT: Alanine aminotransferase
AST: Aspartate aminotransferase
AUROC: Area under the ROC curve
BMI: Body mass index
CI: Confidence interval
CLD: Chronic liver disease
cT1: Corrected T1
HAI: Histological fibrosis stage and activity index
kPa: Kilopascal
MRE: Magnetic resonance elastography
MRE-LS: Magnetic resonance elastography-measured liver stiffness
MRE-SS: Magnetic resonance elastography-measured spleen stiffness
NAFLD: Nonalcoholic fatty liver disease
NASH: Nonalcoholic steatohepatitis
NPV: Negative predictive value
PPV: Positive predictive value
ROC: Receiver operating characteristic
ROI: Region of interest
SWE: Shear wave elastography
SWS: Shear wave speed
