# Supplementary material for: Magnetic resonance elastography in evaluation of liver fibrosis in children with chronic liver disease
Source: Insights Imaging. 2023 Feb 28;14:39. doi: 10.1186/s13244-023-01390-0 (PMC9975132; doi:10.1186/s13244-023-01390-0)
Supplement: Supplementary file 1 — Additional file 1. Correlations between MRE-measured liver and spleen stiffness and clinical, laboratory and other MRE parameters [file 13244_2023_1390_MOESM1_ESM.pdf]

ELECTRONIC SUPPLEMENTARY MATERIAL

Magnetic Resonance Elastography In Evaluation Of Liver Fibrosis in Children with Chronic Liver Disease

Supplementary Table. Correlations between MRE-measured liver and spleen stiffness and clinical, laboratory and other MRE parameters

		MRE-LS	MRE-SS	Ishak stage	HAI	Hepatic steatosis %	AST	ALT	T1	T2	T2*	Corrected T1	R2*
MRE-LS	Correlation Coefficient	1.000	.297*	.553**	.406**	-.330*	.364**	.246	.172	.185	.186	.241	-.326*
	Sig. (2-tailed)	.	.038	.000	.003	.018	.008	.079	.222	.189	.188	.085	.020
	N	52	49	52	52	51	52	52	52	52	52	52	51
MRE-SS	Correlation Coefficient	.297*	1.000	.357*	.077	-.192	.088	.133	-.048	-.014	.218	.108	-.172
	Sig. (2-tailed)	.038	.	.012	.601	.190	.550	.364	.742	.926	.132	.459	.242
	N	49	49	49	49	48	49	49	49	49	49	49	48
Ishak stage	Correlation Coefficient	.553**	.357*	1.000	.485**	-.178	.267	.143	.098	.133	.163	.158	-.223
	Sig. (2-tailed)	.000	.012	.	.000	.210	.055	.312	.489	.345	.248	.265	.116
	N	52	49	52	52	51	52	52	52	52	52	52	51
HAI	Correlation Coefficient	.406**	.077	.485**	1.000	-.148	.312*	.246	.194	.405**	.259	.291*	-.343*
	Sig. (2-tailed)	.003	.601	.000	.	.299	.025	.079	.168	.003	.063	.036	.014
	N	52	49	52	52	51	52	52	52	52	52	52	51
Hepatic steatosis %	Correlation Coefficient	-.330*	-.192	-.178	-.148	1.000	.098	.083	.293*	-.178	-.411**	-.045	.577**
	Sig. (2-tailed)	.018	.190	.210	.299	.	.495	.564	.037	.211	.003	.754	.000
	N	51	48	51	51	51	51	51	51	51	51	51	51
AST	Correlation Coefficient	.364**	.088	.267	.312*	.098	1.000	.833**	.371**	.071	.024	.273*	-.027
	Sig. (2-tailed)	.008	.550	.055	.025	.495	.	.000	.007	.615	.866	.050	.848
	N	52	49	52	52	51	52	52	52	52	52	52	51
ALT	Correlation Coefficient	.246	.133	.143	.246	.083	.833**	1.000	.308*	.114	.031	.232	-.005
	Sig. (2-tailed)	.079	.364	.312	.079	.564	.000	.	.026	.422	.830	.097	.974
	N	52	49	52	52	51	52	52	52	52	52	52	51
T1	Correlation Coefficient	.172	-.048	.098	.194	.293*	.371**	.308*	1.000	.351*	.023	.654**	-.157

	Sig. (2-tailed)	.222	.742	.489	.168	.037	.007	.026	.	.011	.873	.000	.271
	N	52	49	52	52	51	52	52	52	52	52	52	51
T2	Correlation Coefficient	.185	-.014	.133	.405**	-.178	.071	.114	.351*	1.000	.389**	.455**	-.518**
	Sig. (2-tailed)	.189	.926	.345	.003	.211	.615	.422	.011	.	.004	.001	.000
	N	52	49	52	52	51	52	52	52	52	52	52	51
T2*	Correlation Coefficient	.186	.218	.163	.259	-.411**	.024	.031	.023	.389**	1.000	.679**	-.864**
	Sig. (2-tailed)	.188	.132	.248	.063	.003	.866	.830	.873	.004	.	.000	.000
	N	52	49	52	52	51	52	52	52	52	52	52	51
Corrected T1	Correlation Coefficient	.241	.108	.158	.291*	-.045	.273*	.232	.654**	.455**	.679**	1.000	-.639**
	Sig. (2-tailed)	.085	.459	.265	.036	.754	.050	.097	.000	.001	.000	.	.000
	N	52	49	52	52	51	52	52	52	52	52	52	51
R2*	Correlation Coefficient	-.326*	-.172	-.223	-.343*	.577**	-.027	-.005	-.157	-.518**	-.864**	-.639**	1.000
	Sig. (2-tailed)	.020	.242	.116	.014	.000	.848	.974	.271	.000	.000	.000	.
	N	51	48	51	51	51	51	51	51	51	51	51	51

*. Correlation is significant at the 0.05 level (2-tailed).

**. Correlation is significant at the 0.01 level (2-tailed).

†MRE-LS: Magnetic resonance elastography-measured liver stiffness

‡MRE-SS: Magnetic resonance elastography-measured spleen stiffness
